# Insights into Alpha-Hemolysin (Hla) Evolution and Expression among *Staphylococcus aureus* Clones with Hospital and Community Origin

**DOI:** 10.1371/journal.pone.0098634

**Published:** 2014-07-17

**Authors:** Ana Tavares, Jesper B. Nielsen, Kit Boye, Susanne Rohde, Ana C. Paulo, Henrik Westh, Kristian Schønning, Hermínia de Lencastre, Maria Miragaia

**Affiliations:** 1 Laboratory of Molecular Genetics, Instituto de Tecnologia Química e Biológica (ITQB), Oeiras, Portugal; 2 Dept. of Clinical Microbiology 445, Copenhagen University Hospital, Hvidovre, Denmark; 3 Molecular Microbiology of Human Pathogens, ITQB, Oeiras, Portugal; 4 Faculty of Health and Medical Sciences, University of Copenhagen, Copenhagen, Denmark; 5 Laboratory of Microbiology and Infectious Diseases, The Rockefeller University, New York, New York, United States of America; 6 Laboratory of Bacterial Evolution and Molecular Epidemiology, ITQB, Oeiras, Portugal; University of Edinburgh, United Kingdom

## Abstract

**Background:**

Alpha-hemolysin (Hla) is a major virulence factor in the pathogenesis of *Staphylococcus aureus* infection, being active against a wide range of host cells. Although *hla* is ubiquitous in *S. aureus*, its genetic diversity and variation in expression in different genetic backgrounds is not known. We evaluated nucleotide sequence variation and gene expression profiles of *hla* among representatives of hospital (HA) and community-associated (CA) *S. aureus* clones.

**Methods:**

51 methicillin-resistant *S. aureus* and 22 methicillin-susceptible *S. aureus* were characterized by PFGE, *spa* typing, MLST and SCC*mec* typing. The internal regions of *hla* and the *hla* promoter were sequenced and gene expression was assessed by RT-PCR.

**Results:**

Alpha-hemolysin encoding- and promoter sequences were diverse, with 12 and 23 different alleles, respectively. Based on phylogenetic analysis, we suggest that *hla* may have evolved together with the *S. aureus* genetic background, except for ST22, ST121, ST59 and ST93. Conversely, the promoter region showed lack of co-evolution with the genetic backgrounds. Four non-synonymous amino acid changes were identified close to important regions of *hla* activity. Amino acid changes in the RNAIII binding site were not associated to *hla* expression. Although expression rates of *hla* were in general strain-specific, we observed CA clones showed significantly higher *hla* expression (p = 0.003) when compared with HA clones.

**Conclusion:**

We propose that the *hla* gene has evolved together with the genetic background. Overall, CA genetic backgrounds showed higher levels of *hla* expression than HA, and a high strain-to-strain variation of gene expression was detected in closely related strains.

## Introduction

*Staphylococcus aureus* is a human opportunistic pathogen responsible for a wide range of infections that can vary in its clinical presentation and severity. Methicillin-resistant *S. aureus* (MRSA) emerged in 1960 in the United Kingdom and has been a major problem in hospitals (HA-MRSA) worldwide during the last 40 years; however since the late 1990s, MRSA has been emerging as a leading cause of severe infection also in the community, in individuals without recent health-care contact (CA-MRSA) [1], [2].

CA-MRSA present distinct genetic backgrounds from their hospital counterparts, are more susceptible to antibiotics other than beta-lactams, carry the smallest staphylococcal cassette chromosome *mec* types (SCC*mec* IV or V), and have higher virulence capacity [1], [2], [3]. The underlying reasons behind the enhanced virulence of CA-MRSA appear to be multiple including a different capacity to overcome host cell response [4], different distribution of mobile genetic elements carrying virulence determinants [5], allelic variation in virulence determinants located in the core genome and in mobile genetic elements [6], and different levels of expression and protein production of virulence determinants (alpha-hemolysin, collagen adhesin, staphylokinase, coagulase, lipase, enterotoxins C3 and Q, V8 protease and cysteine protease) [7], [8], [9].

**Figure 1:**
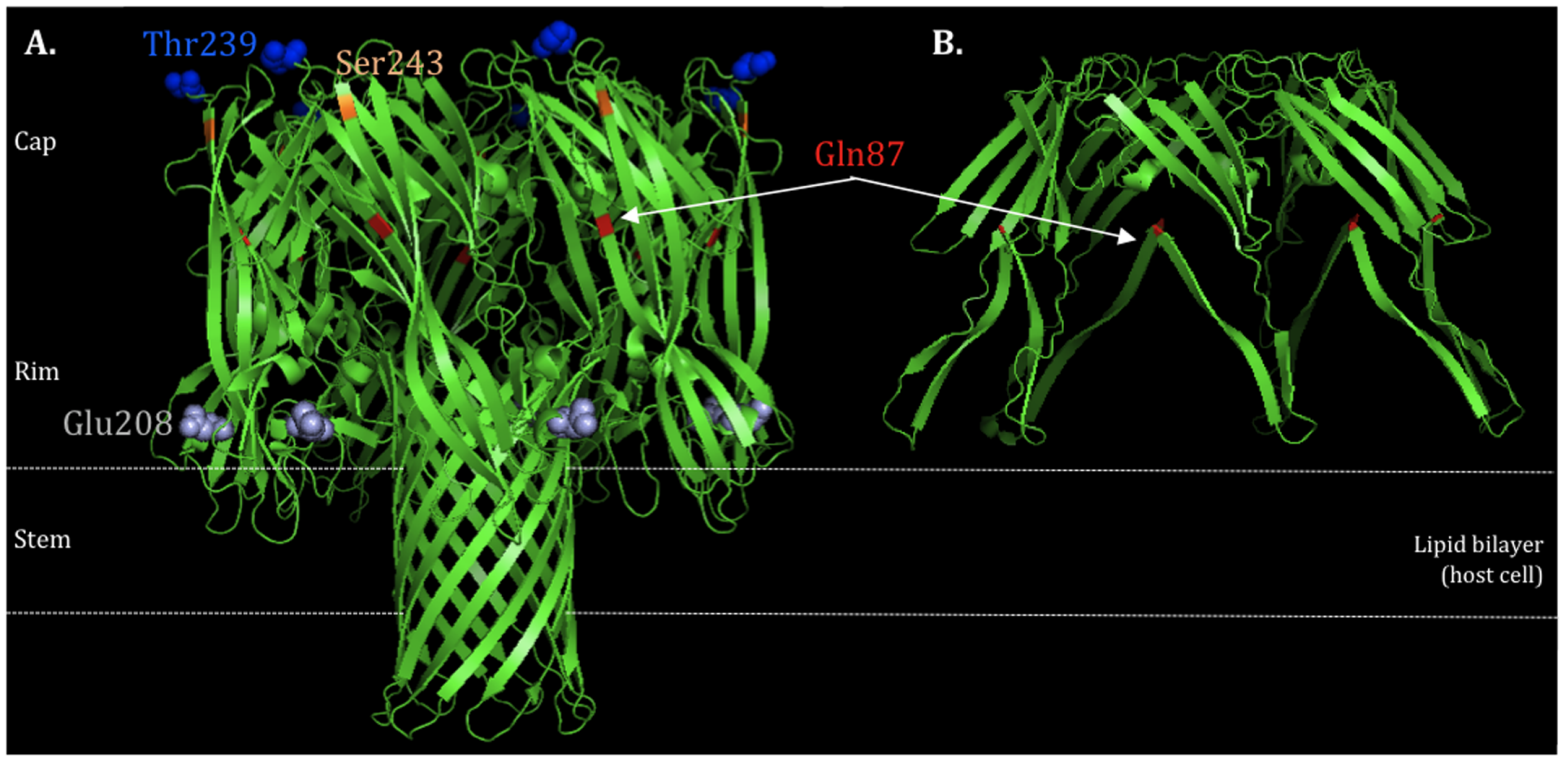
HLA protein structure. A) wildtype (highlighted the non-synonymous mutations Gln87, Glu208, Thr239 and Ser243) and B) truncated protein due to a stop codon at Gln87. Structure generated by the program PyMOL v.1.6.

The alpha-hemolysin or α-toxin (Hla), is one of the major virulence determinants implicated in the pathogenesis of *S. aureus*, associated to severe skin and soft tissue infections (SSTI), necrotizing pneumonia and even sepsis [10]. Hla is the most prominent *S. aureus* cytotoxin that can act against a wide range of host cells including erythrocytes, epithelial cells, endothelial cells, T cells, monocytes and macrophages [10], [11], [12]. The gene encoding Hla is located in the core genome and is expressed as a water-soluble monomer (33.2 kD) that assembles to form a membrane-bound heptameric β-barrel pore (232.4 kD) on susceptible cells leading to cell death and lysis [11]. The overall structure is mushroom-like, divided into three domains: 1) Cap domain: largely hydrophobic, defining the entry of the pore; 2) Rim domain: underside of the Cap, in close proximity to membrane bilayer; 3) Stem domain: part of the transmembrane channel, forming the membrane-perforating β-barrel pore (Figure 1) [10], [11]. Hla expression is mainly controlled by the global toxin accessory gene regulator (*agr*), via the regulatory effector molecule RNAIII [13]. While *agr* provides the first and most important mechanism of up-regulation of *hla*, expression can also be modulated by other regulators, such as SaeR, SarZ, ArlS [14], [15], [16] (up-regulators) and Rot, SarT [17](down-regulators).

Although polymorphisms in the *hla* promoter region have been described [18], the range of genetic diversity and evolution of this toxin has never been assessed in a large representative *S. aureus* collection. Furthermore, although differences in *hla* expression have been described between community- and hospital-associated MRSA, these studies have been performed with a limited number of CA-MRSA epidemic clones [9], or almost exclusively with representatives of the USA300 clone [19], [20], [21]. To better understand the evolutionary history of *hla* and its importance as a virulence factor for CA-MRSA, in this study we compared the *hla* nucleotide sequence and expression among the major epidemic and minor CA and HA clones, including both MRSA and MSSA strains.

## Materials and Methods

### Ethics Statement

Isolates were obtained from routine diagnostic and were analyzed anonymously and only the isolates, not humans, were studied. All data was collected according to the European Parliament and Council decision for the epidemiological surveillance and control of communicable disease in the European community. Ethical approval and informed consent were for that reason not required.

### Bacterial collection

A total of 73 *S. aureus*, including 51 MRSA and 22 MSSA were analyzed in this study. Strains were collected in 13 different countries (Belgium, Bulgaria, Czech Republic, Denmark, Greece, Netherlands, Portugal, Romania, Spain, Sweden, United Kingdom, USA and Brazil), between 1961 and 2009 from both community (n = 46) and hospital (n = 27). The strains comprised a total of 52 *spa* types and 23 sequence types (STs) (see Table S1).

Strains were defined as belonging to CA or HA clones if they contained the same or related genetic backgrounds as the reference CA-MRSA and HA-MRSA epidemic control strains, based on ST, *spa* type and SCC*mec* (in case of MRSA).

### Media and bacterial growth conditions

Before RT-PCR analysis, strains were grown overnight at 37°C on tryptic soy agar plates (TSA). Bacterial growth experiments were performed by growing bacteria in tryptic Soy Broth (TSB) at 37°C with shake and measuring OD (600 nm) each hour in the follow up automatic incubator Bioscreen C (Oy Growth Curves AB, Helsinki, Finland). Plates of 100-well honeycomb (Oy Growth Curves AB, Helsinki, Finland) were filled with 300 µl/well of overnight culture diluted to OD_600_ = 0.05 in TSB growth medium. Three individual growth experiments (SetC, SetD and SetE) were performed for each strain and named accordingly e.g. HLZ6C, HLZ6D and HLZ6E (see Figure S2.I to III).

### Nucleotide sequence of *hla* and promoter region

Chromosomal DNA was extracted from overnight cultures, using the boiling method (100°C for 10 min followed by centrifuged at 13.000 g for 5 minutes). Two sets of primers were designed to span the most polymorphic regions within the *hla* gene and *hla* promoter (considered as the region located −600 bp from *hla* starting codon), after alignment of sequences available on NCBI for *S. aureus*. One set of primers (Forward: hla-F_CGAAAGGTACCATTGCTGGT; Reverse: hla-R_CCAATCGATTTTATATCTTTC) amplified an internal fragment of the *hla* gene (nt 1170419–1170982, CP000730.1) and the other set (Forward: hlaPro-F_CACTATATTAAAAATACATAC; Reverse: hlaPro-R_GTTGTTACTGAGCTGAC) amplified an internal fragment of the *hla* promoter region (nt 1171289–1171773, CP000730.1) (Figure S3). PCR products were sequenced (Macrogen Europe, Amsterdam, The Netherlands) and sequences were analyzed using SeqMan (DNAstar, Lasergene v9, Madison, WI, USA). To each unique *hla* promoter (P) and gene sequence (*hla*) - allotype - a single Arabic number was attributed (e.g. P1, P2; *hla*1, *hla*2). Gene and promoter sequences were deposited in GenBank (accession numbers KM019547–KM019606; KM019607–KM019674).

### Phylogenetic analysis

Phylogenetic relatedness was analyzed using the MEGA5 v5.05 software (http://www.megasoftware.net/) for gene, promoter region and concatenated sequences obtained from 1) gene with promoter region and 2) seven MLST alleles from the 23 representative STs within the collection. Phylogenetic trees were constructed using the Neighbor-Joining clustering method, and 1000 bootstrap replicates, which assigns confidence values for the groupings in the tree.

Moreover, nucleotide diversity (ND) between the two clusters was calculated based on the estimation of the average evolutionary divergence over sequence pairs within the two groups, where the number of base substitutions per site from averaging over all sequence pairs within each group are compared using the maximum composite likelihood model [22].

### Detection of recombination

Alignments from the *hla* gene, *hla* promoter and internal fragments of each of the seven MLST gene were screened for the occurrence of putative recombination events using Recombination Detection Program version 4 (RDP4) (http://web.cbio.uct.ac.za/) with the default settings (with highest acceptable probability value of 0.05). Identification of recombinant sequences recombination breakpoints and major parent was determined using simultaneously nine recombination detection methods (RDP, BOOTSCAN, GENECONV, MAXCHI, CHIMAERA, SISCAN, PhylPro, LARD and 3SEQ. The “minor parent” is considered a sequence closely related to that from which sequences in the proposed recombinant region may have been derived (the presumed donor). The “major parent” was considered as a sequence closely related to that from which the greater part of the recombinant’s sequence may have been derived.

### RT-PCR analysis

Culture growth was stopped at late exponential phase, when alpha-toxin is described to have maximal activity [23], corresponding to the time-points 1) 3 hours 30 min in one group (65 strains) and 2) 4 hours 30 min in another (8 strains). Total RNA was extracted from three biological replicates. Cells were mechanically disrupted with FastPrep-24 Instrument (MP Biomedicals, Solon, OH, USA) and RNA was protected using RNA Protect (Qiagen, Valencia, USA). RNA was extracted automatically using the QIAsymphony platforms (Qiagen, Valencia, USA) with QIAsymphony RNA kit (Qiagen, Valencia, USA).

The RT-PCR assay was performed on a 7500 Real-Time PCR System (Applied Biosystems, Foster City, CA) using the following primers and TaqMan probes: Hla RT_F: TAATGAATCCTGTCGCTAATGCC; HlaRT_R: CACCTGTTTTTACTGTAGTATTGCTTCC; Hla RT Probe: 6FAM-AAACCGGTACTACAGATAT-MGBNFQ. The RT-PCR reaction was performed using the EZ RT-PCR Core Reagents (Applied Biosystems, Foster City, USA), in which RNA is reverse transcribed and amplified in a single reaction. The following PCR protocol was used: 50°C for 2 min, 60°C for 30 min, 95°C for 5 min, followed by 42 cycles of 95°C for 20 sec and 62°C for 1 min. The 16S gene was used as internal or reference control. The primers used for 16S RNA amplification were those previously described [24].

### RT-PCR data analysis

The relative *hla* gene expression was calculated based on the C_t_ (RT-PCR output) of the gene of interest (C_t_ hla) as compared to the C_t_ of the internal control (C_t_ 16S) as follows: Delta C_t_ = C_t_ hla- C_t_ 16S. The lower the Delta C_t_ the higher is the amount of *hla* mRNA and the more the gene is expressed. The reproducibility of the assay was evaluated by the calculation of the arithmetic mean of the relative expression of the three biological replicates (Mean Delta C_t1–3_ =  Average (Delta C_t1_; Delta C_t2_; Delta C_t3_). The reproducibility of RT-PCR reaction was evaluated by the calculation of the standard deviation (STDEV) of Delta C_t_ obtained for each biological replica (Delta C_t1_; Delta C_t2_; Delta C_t3_). Values were considered valid when at least two C_t_ readings exist with STDEV<2.

### Protein structure visualization (pyMOL)

The protein structure was modeled using PyMOL v.1.6 (http://www.pymol.org/) if a nucleotide mutation gave rise to a stop codon.

### Statistical analysis

The statistical analysis was performed using the Graphpad Prism 6 (http://www.graphpad.com/scientific-software/prism/), with the two-tailed Student’s t-test to determine whether the differences of mean expression rates (MSSA *versus* MSSA; HA backgrounds *versus* CA backgrounds) were statistically significant (p≤0.05).

Regression tree analysis was used to explore which variables could be related with the *hla* expression [25]. Trees explain the variation of a single response variable (in this study the *hla* mRNA expression) by repeatedly splitting the data into more homogeneous groups, using combinations of explanatory variables (in our case, the ST, *spa* type, MRSA, MSSA and the type of SCC*mec*).

## Results

### Analysis of polymorphisms in the *hla* gene and *hla* promoter

The sequence analysis of the internal region of *hla* and the *hla* promoter region among the 73 strains identified a total of 12 *hla* and 23 promoter region different sequences (allotypes) (Table 1). We obtained no amplification products for *hla* and *hla* promoter region in one and 13 strains, respectively, which probably result from misparing of the primers used.

**Table 1:** Summary of molecular characterization, sequence variation and relative expression rates of *S. aureus* strains collection.

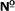	Isolate ID	SCC*mec*	*spa* type	MLST	Branch^1^	Promotor Allotype	Gene Allotype	Nonsynonymous Mutation	Hla Expression (Mean Delta C_t_)^2^	Stddev Delta C_t_ 3	Expression (High/Low)
1	HLZ6	II	t002	**ST5**	L	P4	hla1	D208E	8.69	2*	Low
2	BK2464	II	t002	**ST5**	L	nt	hla1	D208E	5.37	1	**High**
3	HBR73	II	t067	**ST5**	L	P5	hla1	D208E	8.75	1	Low
4	C013	VI	t002	**ST5**	L	P3	hla1	D208E	6.84	1	Low
5	HDES26	VI	t062	**ST5**	L	P3	hla1	D208E	8.01	1	Low
6	HDE288	VI	t311	**ST5**	L	P3	hla1	D208E	6.67	1	Low
7	HSA29	–	t002	**ST5**	L	P3	hla1	D208E	Not Valid	–	Not Valid
8	HDE461	IV	t022	**ST22**	H	P10	hla12	S239T; T243S	6.60	1	Low
9	HAR22	IV	t022	**ST22**	H	P11	hla13	S239T; T243S	6.43	1	Low
10	HSMB280	IV	t032	**ST22**	H	P10	hla12	S239T; T243S	4.71	1	**High**
11	LBM12	IV	t747	**ST1806**	H	nt	hla12	S239T; T243S	9.28	1	Low
12	HSMB184	–	t5951	**ST1806**	H	P10	hla12	S239T; T243S	6.74	1	Low
13	HPH2	II	t018	**ST36**	H	P7	hla8	D208E; S239T; stop codon	8.02	2*	Low
14	HAR24	II	t018	**ST36**	H	nt	hla8	D208E; S239T; stop codon	9.62	2*	Low
15	DEN4415	II	t021	**ST36**	H	P7	hla8	D208E; S239T; stop codon	8.95	2*	Low
16	C563	IV	t015	**ST45**	H	nt	hla10	S239T	7.02	1	Low
17	C036	V	t015	**ST45**	H	nt	hla10	S239T	6.24	0	Low
18	HAR38	IV	t004	**ST45**	H	P7	hla10	S239T	10.38	1	Low
19	HFX77	III	t037	**ST239**	L	P1	hla4	–	8.74	2*	Low
20	HUC343	IIIA	t037	**ST239**	L	P1	hla4	–	8.27	0	Low
21	HU25	IIIA	t138	**ST239**	L	P1	hla4	–	8.17	1	Low
22	BK1953	IA	t051	**ST247**	L	P1	hla4	–	7.71	1	Low
23	HPV107	IA	t051	**ST247**	L	P1	hla4	–	7.56	0	Low
24	HSJ419	IA	t725	**ST247**	L	P1	hla4	–	8.23	1	Low
27	E2125	I	t051	**ST247**	L	P1	hla4	–	7.29	0	Low
25	10395	I	t008	**ST250**	L	P2	hla4	–	8.15	1	Low
26	COL	I	t008	**ST250**	L	P1	hla4	–	8.01	1	Low
28	HFX74	IV	t008	**ST8**	L	P1	hla4	–	6.46	1	Low
29	USA300	IV	t008	**ST8**	L	P1	hla4	–	6.19	3*	Low
30	C438	IV	t024	**ST8**	L	P1	hla4	–	6.07	1	Low
31	C574B	IV	t1257	**ST612**	L	P1	hla4	–	Not Valid	–	Not Valid
32	LBM27	–	t024	**ST8**	L	P1	hla4	–	8.12	0	Low
33	LBM74	–	t008	**ST8**	L	P1	hla4	–	5.87	1	Low
34	C270	IV	t1381	**ST1**	L	P17	hla2	–	8.81	1	Low
35	USA400	IV	t127	**ST1**	L	P17	hla2	–	6.01	2*	Low
36	LBM36	–	t127	**ST1**	L	P18	hla2	–	11.09	1	Low
37	C577	IV	t216	**ST59**	L	P20	hla5	–	5.35	0	**High**
38	C583	IV	t437	**ST59**	L	P19	hla5	–	5.31	1	**High**
39	C434	V	t437	**ST59**	L	P19	hla5	–	9.14	1	Low
40	C018	IV	t1819	**ST93**	L	nt	hla7	–	5.16	1	**High**
41	C491	IV	t202	**ST93**	L	P21	hla7	–	5.45	0	**High**
42	LBM54	IV	t011	**ST398**	H	P12	hla11	–	4.46	2*	**High**
43	C482	IV	t011	**ST398**	H	P13	hla11	–	3.25	1	**High**
44	C496	VII	t108	**ST398**	H	nt	hla11	–	2.85	1	**High**
45	LBM40	–	t034	**ST398**	H	P12	hla11	–	5.37	1	**High**
46	C017	IV	t019	**ST30**	H	nt	hla9	D208E; S239T	4.53	0	**High**
47	C385	IV	t019	**ST30**	H	P7	hla9	D208E; S239T	7.25	1	Low
48	C479	IV	t019	**ST30**	H	nt	hla9	D208E; S239T	8.10	1	Low
71	HUC585	–	t342	**ST30**	H	P7	hla9	D208E; S239T	5.14	1	**High**
69	HFF204	–	t318	**ST30**	H	P9	hla9	D208E; S239T	6.23	1	Low
70	HFA30	–	t012	**ST30**	H	P8	hla8	D208E; S239T; stop codon	7.94	1	Low
49	HSJO7	IV	t148	**ST72**	L	P14	hla1	D208E	6.56	1	Low
50	USA700	IV	t148	**ST72**	L	P14	hla1	D208E	5.76	0	Low
51	COO3	IV	t791	**ST72**	L	P15	hla1	D208E	6.28	1	Low
52	SAMS1024	IV	t1346	**ST1810**	L	P14	hla1	D208E	4.78	1	**High**
53	HUC594	–	t148	**ST72**	L	P14	hla1	D208E	8.36	1	Low
54	HFA28	–	t126	**ST72**	L	P14	hla1	D208E	4.56	2*	**High**
55	C238	–	t3682	**ST72**	L	P14	hla1	D208E	4.64	1	**High**
56	C168	IV	t044	**ST80**	L	P16	hla1	D208E	8.20	0	Low
57	C485	IV	t044	**ST80**	L	P16	hla1	D208E	5.72	1	**High**
58	C014	IV	t131	**ST80**	L	P16	hla1	D208E	4.87	0	**High**
59	LBM25	–	t1509	**ST15**	L	P2	hla1	D208E	6.69	0	Low
60	C157	–	t084	**ST15**	L	P2	hla1	D208E	4.86	1	**High**
61	C230	–	t346	**ST15**	L	P2	hla1	D208E	9.03	2*	Low
62	HBA33	–	t258	**ST25**	L	P6	hla1	D208E	5.73	1	**High**
63	C095	–	t2909	**ST25**	L	P6	hla1	D208E	4.16	1	**High**
64	C141	–	t081	**ST25**	L	P6	hla1	D208E	4.50	2*	**High**
65	HBA34	IV	t308	**ST121**	L	nt	hla6	–	5.62	1	High
66	HUC574	–	t435	**ST121**	L	P1	hla6	–	5.19	1	**High**
67	HUC587	–	t159	**ST121**	L	P2	hla6	–	5.09	1	**High**
68	HUC578	–	t284	**ST121**	L	P1	hla6	–	7.10	2*	Low
72	LBM23	–	t100	**ST9**	L	P22	hla1	D208E	5.48	2*	**High**
73	HFX84	–	t267	**ST97**	L	P23	hla3	–	9.03	1	Low

1 H: High polymorphism; L: Low polymorphism;

2 Mean Delta C_t1–3_ =  Average (Delta C_t1_; Delta C_t2_; Delta C_t3_), Delta C_t_ = C_t_ hla−C_t_ 16S; Not valid: only one C_t_ reading;

3 *low reproducibility between three C_T_ values (Stddv≤2). nt: non typable; Stddv: standard deviation.

From the 12 *hla* (*hla*1–12), we observed that only a single *hla-*allotype was found among representatives of a specific ST, except for ST22 (*hla*12; *hla*13) and ST30 (*hla*8; *hla*9) where two different alleles were identified. On the other hand, the most frequent alleles, *hla1* (33.3%, n = 24) and *hla4* (20.8%, n = 15), were identified in more than one ST.

Regarding the nucleotide changes identified in the *hla*, some correspond to non-synonymous mutations (E208, T239 and S243) and, in one particular case, to a stop codon (Table 1 and 2). The substitutions observed did not correspond to any difference in the charge or polarity of the amino acid (aa). However, changes in molecular weight were observed: i) changes from aa D208 to aa E208 (D208E) and from aa S239 to T239 (S239T) gave rise to a higher molecular weight aa; and ii) change from aa T243 to S243 (T243S) resulted in a lower molecular weight aa; of note all changes occurred in the Rim domain of the protein. In a particular case, the aa change gave rise to a stop codon located in the CAP domain, in strains of ST36. Protein structure modeling showed that a protein of about one third of its real size is produced, truncated at the Gln87 (Figure 1, A and B). The truncation is in the outside part of the domain, suggesting that this will affect the capacity of the Hla to form cell wall pores, and ultimately to induce hemolysis.

**Table 2:** Strains data distribution based on promoter allotypes.

		Promotor allotype	Gene allotype	Non Synonymous Mutation	Isolates Molecular Characterization	Expression Category
**CA** backgrounds	**ST398**	P13	hla11	–	ST398-IV, t011	**High expression**
		P12			ST398, t034	**High expression**
		NT			ST398-VII, t108	**High expression**
		P12			ST398-IV, t011	High expression*
	**ST25**	P6	hla1	D208E	ST25, t258	**High expression**
					ST25, t081	High expression*
					ST25, t2909	**High expression**
	**ST9**	P22	hla1	D208E	ST9, t100	High expression*
	**ST93**	P21	hla7	–	ST93-IV, t202	**High expression**
		NT			ST93-IV, t1819	**High expression**
	**ST121**	P2	hla6	–	ST121, t159	**High expression**
		P1			ST121, t435	**High expression**
		NT			ST121-IV, t308	**High expression**
		P1			ST121, t284	Low expression*
	**ST72**	P14	hla1	D208E	ST72-IV, t148	**High expression**
		P14			ST72, t3682	**High expression**
		P14			ST1810-IV, t1346	**High expression**
		P14			ST72, t126	High expression*
		P15			ST72-IV, t791	Low expression
		P14			ST72-IV, t148	Low expression
		P14			ST72, t148	Low expression
	**ST80**	P16	hla1	D208E	ST80-IcV, t131	**High expression**
					ST80-IV, t044	**High expression**
					ST80-IV, t044	Low expression
	**ST30**	P7	hla9	D208E; S239T	ST30, t342	**High expression**
		NT			ST30-IV, t019	**High expression**
		P7			ST30-IV, t019	Low expression
		P9			ST30, t318	Low expression
		NT			ST30-IV, t019	Low expression
		P8	hla8	D208E; S239T; stop codon	ST30, t012	Low expression
	**ST15**	P2	hla1	D208E	ST15, t084	**High expression**
					ST15, t346	Low expression*
					ST15, t1509	Low expression
	**ST59**	P20	hla5	–	ST59-IV, t216	**High expression**
		P19			ST59-IV, t437	Low expression
		P19			ST59-V, t437	Low expression
	**ST1**	P17	hla2	–	ST1-IV, t1381	Low expression
		P17			ST1-IV, t127	Low expression*
		P18			ST1, t127	Low expression
	**ST8**	P1	hla4	–	ST8-IV, t008	Low expression
					ST8-IV, t024	Low expression
					ST8-IV, t008	Low expression*
					ST8, t008	Low expression
					ST612-IV, t1257	Not valid**
					ST8, t024	Low expression
	**ST97**	P23	hla3	–	ST97, t267	Low expression
**HA** backgrounds	**ST22**	P10	hla13	S239T; T243S	ST22-IV, t032	**High expression**
		P10	hla12		ST22-IV, t022	Low expression
		P11			ST22-IV, t022	Low expression
		P10			ST1806, t5951	Low expression
		NT			ST1806-IV, t747	Low expression
	**ST5**	NT	hla1	D208E	ST5-II, t002	**High expression**
		P3			ST5-VI, t002	Low expression
		P3			ST5-VI, t062	Low expression
		P3			ST5-VI, t311	Low expression
		P4			ST5-II, t002	Low expression*
		P3			ST5, t002,	Not valid**
		P5			ST5-II, t067	Low expression
	**ST36**	P7	hla8	D208E; S239T; stop codon	ST36-II, t018	Low expression*
		P7			ST36-II, t021	Low expression*
		NT			ST36-II, t01	Low expression*
	**ST45**	NT	hla10	S239T	ST45-IV, t015	Low expression
		NT			ST45-V, t015	Low expression
		P7			ST45-IV, t004	Low expression
	**ST239**	P1	hla4	–	ST239-IIIA, t037	Low expression
					ST239-III, t037	Low expression*
				–	ST239-IIIA, t138	Low expression
	**ST247**	P1	hla4	–	ST247-I, t051	Low expression
					ST247-IA, 051	Low expression
					ST247-IA, t051	Low expression
					ST247-IA, t725	Low expression
	**ST250**	P1	hla4	–	ST250-I, t008	Low expression
		P2			ST250-I, t008	Low expression

(*)(**) relative expression values not valid (SDV≤2 or only one C_T_ reading).

A high number of sequence variations were identified in the *hla* promoter region, (n = 23) (P1–23) (Table 1 and 2). Although we found that some STs were associated to a specific promoter allotype, and some promoters were identified in a single ST, we also identified cases where single STs were associated to different promoters (8 out of 23) and examples in which a single promoter allotype was associated to different STs (5 out of 23). This is the case of the most frequent promoter (P1) that was found in about one third of the strains analyzed (25.4%, n = 16), including several different STs.

A particular highly polymorphic region corresponding to nt −22 to −24 from the start codon, was found in the majority (16 out of 23) of the promoter allotypes (exceptions P1, P6, P13, P14, P15, P18 and P23). These polymorphisms are located in the vicinity of RNAIII binding site [26]; however, we could not find a direct correlation between a particular nucleotide sequence and a specific expression pattern (high or low expression). For example, the sequence T**T**T, observed in two strains belonging to ST398 that have a high level expression, was also observed in strains with low expression belonging to other genetic backgrounds (ST8, ST239, ST247, ST250, ST36, ST45 and ST22).

### Alpha-hemolysin evolutionary history

**Figure 2:**
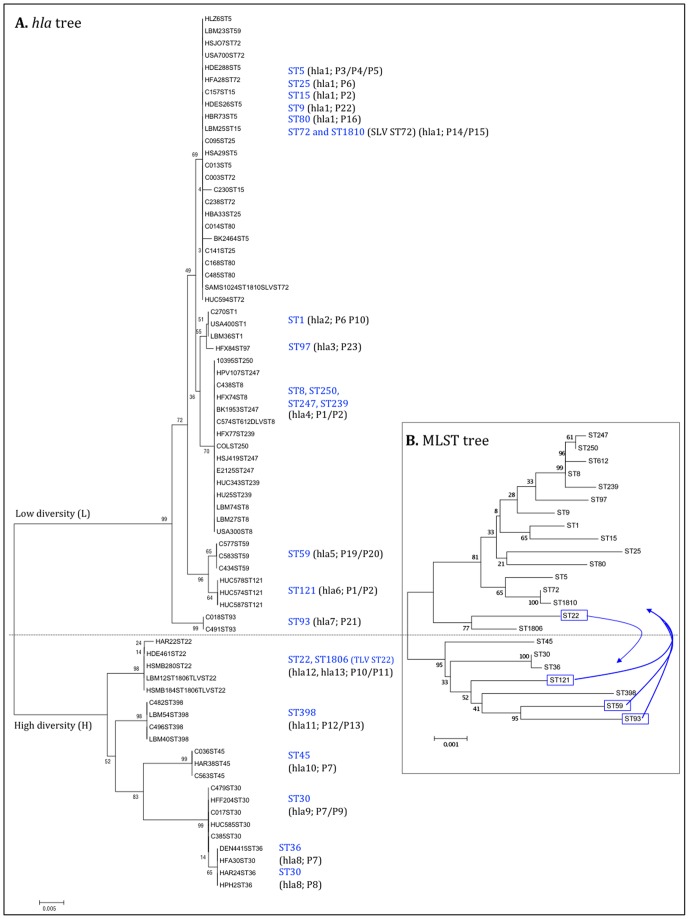
Phylogenetic trees of *hla* gene (A) and concatenated sequences of MLST alleles (B) from 23 STs representatives of the strains collection. The tree was constructed using MEGA 5 with Neighbour-joining method and bootstrap values provided as percents over 1000 replications. Branch length values are indicated and the percentage of replicate trees (bootstrap test) are shown next to the branches. The dashed line indicates the separation of the two evolutionary branches.

In order to better understand the evolution of *hla* gene within the *S. aureus* population, we constructed phylogenetic trees from the *hla* and *hla* promoter sequences, separately or concatenated (Figure 2, A) and compared it with the tree constructed from the concatenated sequences of the seven housekeeping genes used in MLST, including all the STs represented in the strain collection described here (Figure 2, B).

The phylogenetic tree constructed for the *hla* gene showed two distinct major clusters with different evolutionary clocks that differed in their nucleotide diversity (ND, see Materials and Methods): cluster (L) with lower diversity (ND = 0.005), and cluster H with higher diversity (ND = 0.019). Cluster L included more than 70% of strains (71.2%, n = 52), and five sub-clusters; Cluster H contained about 29% of the strains (28.8%, n = 21), and comprised four minor sub-clusters including *hla8*–*hla12* alleles, which were found in strains of ST30, ST36, ST45, ST398 and ST22.

As opposed to the phylogenetic tree constructed from *hla* gene, the one constructed from the promoter region did not show two distinct evolutionary branches (Figure S1). Moreover, dissimilar subgroup clustering was noticed in the tree constructed from the promoter gene sequence. For example, ST45, ST30 and ST36 backgrounds were clustered together in the promoter sequence-based tree whereas in the *hla* sequence-based tree ST45 was placed separately from ST30 and ST36 cluster (branch H). The same type of observations can be drawn for most of STs. Overall the promoter region showed to be more diverse than the *hla* gene sequence among the different backgrounds.

On the other hand, when we compared the phylogenetic tree constructed with the *hla* gene with that constructed from MLST concatenated genes, the same type of division into two distinct main clusters was observed (Figure 2). Moreover, the majority of STs were equally distributed between the two clusters in the two trees. The only exceptions were ST22, ST121, ST59 and ST93 that in the two trees have exchanged their positions from one cluster to the other (Figure 2, B-blue arrows).

### Detection of recombination in *hla* gene, h*la* promoter and MLST genes

To understand if recombination could explain the incongruence found between the trees constructed from *hla* and MLST concatenated genes, we screened the *hla* gene, *hla* promoter and each MLST gene for recombination events using the RDP4 software.

The SiScan and 3Seq methods detected one recombination event in the *hla* gene. This event corresponded to a fragment ending in positions 385–410 of the *hla* alignment, however the beginning breakpoint was not possible to determine. In the collection analyzed this event was detected in five isolates belonging to ST22 or related STs (HSMB280, HDE461, HAR22 and LBM12 (TLV ST22) and HSMB184 (TLV ST22)) and four isolates of ST398 (LBM54, LBM40, C496, C482_ST398). The ST30 HFF204 strain was identified as the minor parent (97.8% identity with ST22 strains and −99.3% identity with ST398 strains) and ST121 strain HUC587 was identified as the major parent (with 100% identity to ST398 strains and 93.5–95.2% identity with ST22 strains) of the recombining fragment. A trace signal of recombination of this same event was also identified among ST45 isolates; however this signal was not statistically significant. Interestingly all the recombination events were detected in strains belonging to the high genetic diversity cluster in the tree constructed from *hla* gene. In the *hla* promoter region no recombination events were detected.

We have performed the same type of analysis using the internal sequences of each of the seven housekeeping used in MLST scheme, including the alleles present in all STs identified in this study, however no recombination events were detected in any of the genes.

Altogether the data gathered suggest that for the majority of strains *hla* gene evolved together with the genetic background. The different clustering of ST22 and ST121 strains, in the trees constructed from MLST concatenated genes and *hla* gene, may derive from recombination events occurring in the *hla* gene. Similarly these type of events might explain the genetic diversity observed in cluster H in the *hla* tree in strains belonging to ST22, ST398, ST45, ST30 and ST36 (H cluster of *hla* tree).

### Expression of alpha-hemolysin

The expression of alpha-hemolysin in the 73 strains was assessed by RT-PCR, in three biological replicates. Fifteen of the 73 strains (20.5%) were excluded from the final analysis, either because a single valid determination for Delta C_t_ (N = 2) was obtained or because C_T_ obtained from the different biological replicates were not reproducible (N = 13).

**Figure 3:**
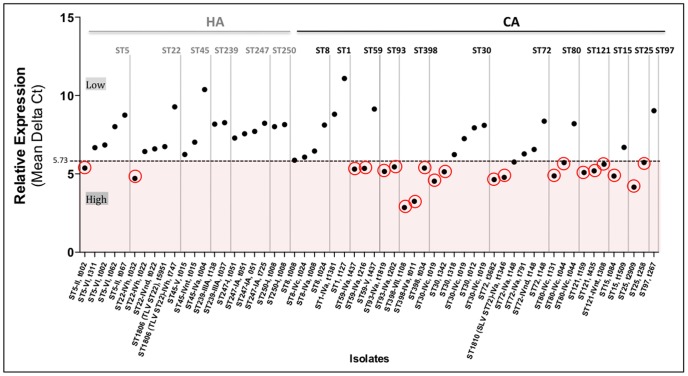
HA and CA strains relative expression distribution. Mean of expression rates from three biological replicates. Dashed line corresponding to the mean Ct value 5.73 results from the regression tree analysis which split strains in two distinct groups, at *spa* type level: a) high expression group - corresponding to strains with Mean Delta Ct≤5.73 and b) low expression group- corresponding to strains with Mean Delta Ct>5.73). Highlighted in red are the high expressing strains.

The analysis of the regression tree split the response variable into two distinct groups, according to the *spa* type of the strains. There was a group of strains with mean Delta C_t1–3_≤5.73, that was classified as a high expression group and a second group with a mean Delta C_t1–3_>5.73 classified as a low expression group (Table 1, Table 2 and Figure 3). Overall the regression tree explained 60% of the variance in the data. This is mostly because there were strains expressing a low or high mean Delta C_t_ that were classified in the same *spa* type; those were the cases of *spa* types t002, t019, t044 and t437.

Furthermore, we explored in each of the *spa* types what other explanatory variables (ST, MRSA, MSSA and type of SCC*mec*) could differentiate the inclusion of some strains in the low or high expression group, but we found no associations with the variables we measured in the study.

**Figure 4:**
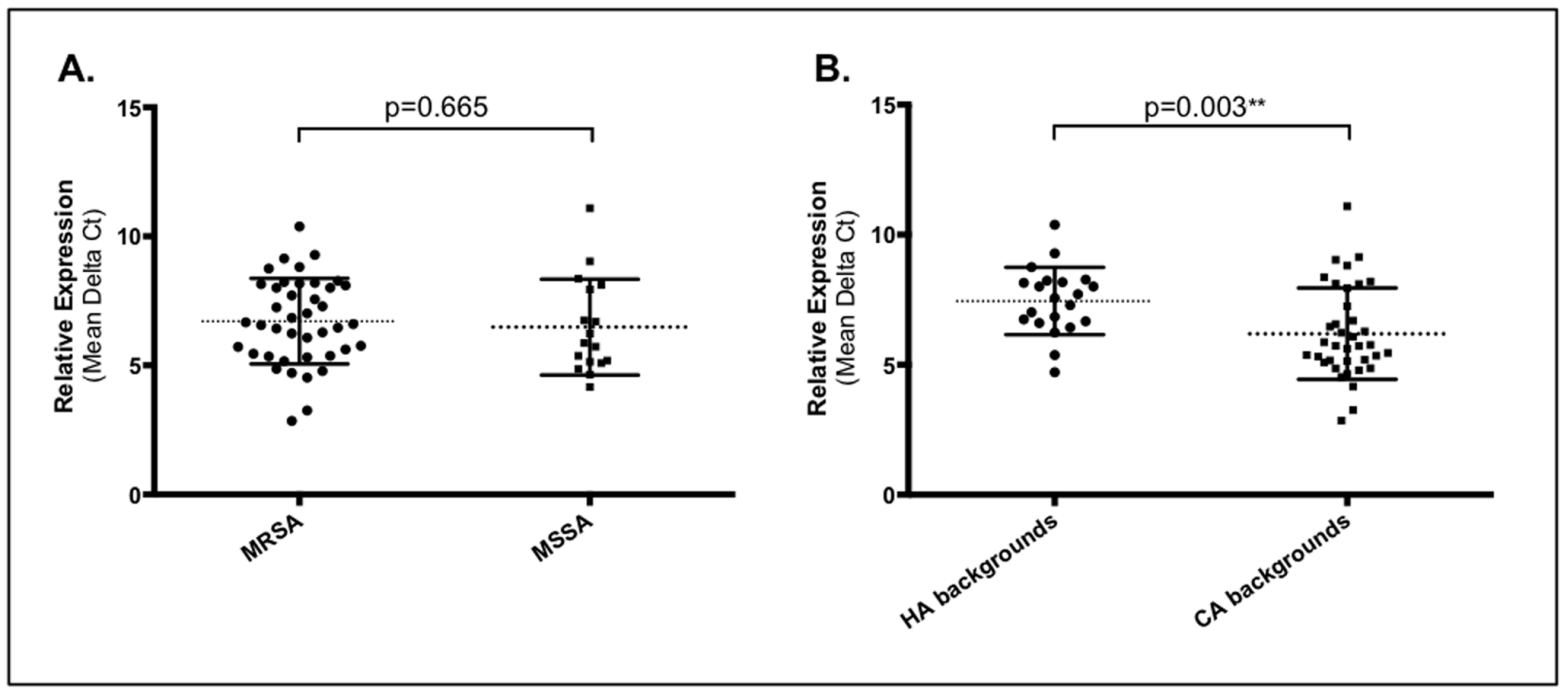
Distribution of the relative *hla* expression. Mean of relative expression of three independent readings. Expression comparison between a) MRSA and MSSA and b) HA and CA backgrounds using the Two-tailed Student’s t-test. Statistically significance (p≤0.05) (**).

We observed that the *hla* expression level varied within strains of the same ST (Figure 3; Table 1 and 2). In fact, in some cases the same ST comprised strains with both high and low levels of expression (ST5, ST15, ST22, ST30, ST59, ST72 and ST80). Moreover, we found that the expression rates did not differ significantly (P = 0.665) between MRSA and MSSA strains. However, we did find a correlation between the *hla* expression and the origin of the genetic backgrounds. Actually, strains of CA genetic backgrounds showed, in general, higher mean expression rates than strains of HA backgrounds (p = 0.003) (Figure 4). Among the 21 strains (36.2%, 21 out of 58) with high expression level, only two (9.5%) belonged to HA backgrounds (ST22-IVh, t032 and ST5-II, t002) whereas the majority (90.5%, n = 19) were represented by CA backgrounds (Table 1 and Table 2). Moreover, two additional CA strains, ST72-IVa-t148 and ST8-MSSA-t008, showed expression rates near the cutoff value (5.73), with 5.76 and 5.87, respectively. These were considered as belonging to the low-level expression group.

The three strains with the highest expression rate were ST398-VII-t108 (2.85), ST398-IVa-t011 (3.25) and ST25-MSSA-t2909 (4.16) and strains with the lowest rate were ST1806 (TLV ST22)-IVh-t747 (9.28), ST45-IVa-t004 (10.38) and ST1-MSSA-t127 (11.09).

We observed that some promoters and gene alleles (P6, P12/P13, P21; and *hla7*, *hla9*, *hla11*) were exclusively associated to a high expression level profile, while others (P3/P4/P5, P7, P8/P9, P11, P15, P17/P18, P23; and *hla4*, *hla8*, *hla10*) were exclusively associated to a low expression level (Table 1 and 2). But we also found promoter and gene allotypes that were associated to both high and low expression levels.

## Discussion

Although Hla is one of the most important *S. aureus* virulence factors [10], to the best of our knowledge, this is the first study in which the variation in *hla* nucleotide sequence and gene expression was assessed in such a large and representative collection.

We found that the nucleotide sequence of *hla* was highly diverse. The high degree of diversity found within *hla* is in accordance to results obtained for other exotoxins, which are generally highly polymorphic [27]. Four non-synonymous substitutions (Q87 stop codon, D208E, S239T and T243S) were identified, that are located in two structural protein domains which are essential for Hla oligomerization and pore formation (Rim and Cap) [11], [28], [29]. The impact of these amino acid (aa) changes on *hla* activity is uncertain. If by one hand, the aa changes described implicate differences in the molecular weight of the aa, that can have influence in the three dimensional structure stability and activity of the protein; on the other hand these aa changes did not match any of the aa previously described to be essential for Hla pore formation.

Furthermore, Walker and Bayley showed that multiple mutations in this same region (residues spanning Hla235–250) did not alter Hla activity in terms of binding, oligomerization or lysis. Thus, it would not be expected that S239T or T243S had significant biological impact in terms of toxin function. The unique mutation with an identified role in Hla function is the stop codon found in the ST36 and ST30 strains that was previously described by DeLeo and co-authors [30] to hinder toxin production and to originate a less virulent strain in a murine infection model. The true effect of the non-synonymous substitutions identified in our study in the activity of the protein would have to be tested by the construction of site directed mutagenesis mutants and by performing binding, oligomerization, hemolysis and *in vivo* models assays.

The construction of phylogenetic trees from the *hla* defined the existence of two clusters with different levels of genetic diversity suggesting that *hla* is evolving at different rates in different genetic backgrounds. Interestingly, the most diverse cluster included the clonal types which are presently more disseminated or that emerged recently (like ST398). This might be related to the fact that these clones still need to evolve to evade the human immune system and not enough time as elapsed for the most adapted allele to have been selected [31]. On the other hand the recombination events detected in the *hla* gene in this study were all in strains belonging to the high genetic diversity cluster, suggesting that this mechanism might have been important in the most recent *hla* evolution and diversification.

Interestingly, the phylogenetic tree constructed from the *hla* gene was similar to that constructed from MLST genes, in the sense that both trees distributed the different STs similarly in two main clusters. This observation suggests that *hla* gene has evolved together with the *S. aureus* genetic background. A similar type of correlation with the genetic background was previously described for adhesins, either located in the core genome (*clfA*, *clfB*, *fnbA*, *map*, *sdrC*, and *spa*) or accessory genome (*ebpS*, *fnbB*, *sdrD*, and *sdrE*) [32]. Although this was the case for the great majority of STs, we observed that four STs (ST22, ST121, ST59, ST93) were located in different clusters in the *hla* and MLST trees. Our results suggest that recombination occurring at the *hla* level, might explain the different clustering of strains belonging to ST22 and ST121. No recombination events were, however, detected in MLST genes or *hla* sequences of strains belonging to ST59 and ST93, suggesting that their displacement in the two trees could derive from different phenomena, like random mutation.

It was previously suggested that CA-MRSA expressed more *hla* than HA-MRSA [9]. Results from our study allowed us to extend this conclusion to virtually all epidemic CA, but also in two particular cases of HA genetic backgrounds. The CA strains belonging to ST398, ST25, ST121 and ST93 showed uniformly high relative expression rates and strains belonging to ST36, ST45, ST239, ST247 and ST250 showed uniformly low expression rates. To understand if in fact these patterns of expression are characteristic of these clones, more strains within each clone should be studied for *hla* expression. Nevertheless, we could not correlate the *hla* expression rate with any particular polymorphism within the promoter or any aa substitution in the *hla* gene. The results suggest that *hla* regulation is probably a result of combination of factors which are redundant, rather than associated to a single genetic event. In fact, it has been demonstrated by several authors that alpha-hemolysin is part of a complex regulatory network, that includes the main two-component systems (TCS) – Agr – that in turn is controlled by a diverse pool of regulatory networks that coordinately interact in response to external stimulus and cell signals, namely others TCS (SaeRS, ArlRS and SrrAB), alternative sigma factors (σ^B^), and transcription factors (e.g. SarS, SarT, Rot, SarA, SarZ) [33], [34].

We showed that *hla* evolved together with the genetic background. Moreover, the most epidemic CA-MRSA genetic backgrounds express more *hla* than the most epidemic HA-MRSA genetic backgrounds. However, the finding of frequent strain-to-strain variation in the expression level of *hla* within strains of the same clonal types suggests that *hla* polymorphisms cannot be used as genetic markers of virulence and investigators should remain cautious when inferring conclusions for the entire MRSA population from studies performed with a limited number of strains.

## Supporting Information

Figure S1 Phylogenetic trees of the *hla* gene, promoter gene and concatenated sequences of both. The tree was constructed using MEGA 5 with Neighbour-joining method and bootstrap values provided as percents over 1000 replications. Branch length values are indicated and the percentage of replicate trees (bootstrap test) are shown next to the branches. The dashed line indicates the separation of the two evolutionary branches (L and H).
(TIF)

Figure S2 **I.** Growth curves for triplicates of each *S. aureus* strain – Set C. **II.** Growth curves for triplicates of each *S. aureus* strain – Set D. **III.** Growth curves for triplicates of each *S. aureus* strain – Set E.
(TIFF)

Figure S3 Internal sequences of *hla* promoter (highlighted blue) and *hla* gene (highlighted orange) used for analysis in this study. Primers used are highlighted. The sequence shown corresponds to the promoter and *hla* regions of USA300 strain from our collection blasted against USA300_TCH1516.
(TIF)

Table S1 Molecular characterization of the 73 MRSA and MSSA strains included in this study [35]–[50].
(DOC)

## References

[1] Deurenberg RH, Stobberingh EE (2009) The molecular evolution of hospital- and community-associated methicillin-resistant *Staphylococcus aureus* . Curr Mol Med 9: 100–115. 19275621 10.2174/156652409787581637

[2] David MZ, Daum RS (2010) Community-associated methicillin-resistant *Staphylococcus aureus*: epidemiology and clinical consequences of an emerging epidemic. Clin Microbiol Rev 23: 616–687. 20610826 10.1128/CMR.00081-09 PMC2901661

[3] Otto M (2013) Community-associated MRSA: What makes them special? Int J Med Microbiol. 10.1016/j.ijmm.2013.02.007 PMC3729626 23517691

[4] Kobayashi SD, Voyich JM, Burlak C, DeLeo FR (2005) Neutrophils in the innate immune response. Arch Immunol Ther Exp (Warsz) 53: 505–517. 16407783

[5] Baba T, Takeuchi F, Kuroda M, Yuzawa H, Aoki K, et al (2002) Genome and virulence determinants of high virulence community-acquired MRSA. Lancet 359: 1819–1827. 12044378 10.1016/s0140-6736(02)08713-5

[6] Diep BA, Gill SR, Chang RF, Phan TH, Chen JH, et al (2006) Complete genome sequence of USA300, an epidemic clone of community-acquired meticillin-resistant *Staphylococcus aureus* . Lancet 367: 731–739. 16517273 10.1016/S0140-6736(06)68231-7

[7] Burlak C, Hammer CH, Robinson MA, Whitney AR, McGavin MJ, et al (2007) Global analysis of community-associated methicillin-resistant *Staphylococcus aureus* exoproteins reveals molecules produced in vitro and during infection. Cell Microbiol 9: 1172–1190. 17217429 10.1111/j.1462-5822.2006.00858.x PMC2064037

[8] Loughman JA, Fritz SA, Storch GA, Hunstad DA (2009) Virulence gene expression in human community-acquired *Staphylococcus aureus* infection. J Infect Dis 199: 294–301. 19115951 10.1086/595982 PMC2843142

[9] Li M, Cheung GY, Hu J, Wang D, Joo HS, et al (2010) Comparative analysis of virulence and toxin expression of global community-associated methicillin-resistant *Staphylococcus aureus* strains. J Infect Dis 202: 1866–1876. 21050125 10.1086/657419 PMC3058913

[10] Berube BJ, Bubeck Wardenburg J (2013) *Staphylococcus aureus* alpha-Toxin: Nearly a Century of Intrigue. Toxins (Basel) 5: 1140–1166. 23888516 10.3390/toxins5061140 PMC3717774

[11] Song L, Hobaugh MR, Shustak C, Cheley S, Bayley H, et al (1996) Structure of staphylococcal alpha-hemolysin, a heptameric transmembrane pore. Science 274: 1859–1866. 8943190 10.1126/science.274.5294.1859

[12] Valeva A, Palmer M, Bhakdi S (1997) Staphylococcal alpha-toxin: formation of the heptameric pore is partially cooperative and proceeds through multiple intermediate stages. Biochemistry 36: 13298–13304. 9341221 10.1021/bi971075r

[13] Novick RP, Ross HF, Projan SJ, Kornblum J, Kreiswirth B, et al (1993) Synthesis of staphylococcal virulence factors is controlled by a regulatory RNA molecule. EMBO J 12: 3967–3975. 7691599 10.1002/j.1460-2075.1993.tb06074.x PMC413679

[14] Ballal A, Ray B, Manna AC (2009) *sarZ*, a *sarA* family gene, is transcriptionally activated by MgrA and is involved in the regulation of genes encoding exoproteins in *Staphylococcus aureus* . J Bacteriol 191: 1656–1665. 19103928 10.1128/JB.01555-08 PMC2648185

[15] Liang X, Yu C, Sun J, Liu H, Landwehr C, et al (2006) Inactivation of a two-component signal transduction system, SaeRS, eliminates adherence and attenuates virulence of *Staphylococcus aureus* . Infect Immun 74: 4655–4665. 16861653 10.1128/IAI.00322-06 PMC1539584

[16] Liang X, Zheng L, Landwehr C, Lunsford D, Holmes D, et al (2005) Global regulation of gene expression by ArlRS, a two-component signal transduction regulatory system of *Staphylococcus aureus* . J Bacteriol 187: 5486–5492. 16030243 10.1128/JB.187.15.5486-5492.2005 PMC1196029

[17] Schmidt KA, Manna AC, Gill S, Cheung AL (2001) SarT, a repressor of alpha-hemolysin in *Staphylococcus aureus* . Infect Immun 69: 4749–4758. 11447147 10.1128/IAI.69.8.4749-4758.2001 PMC98561

[18] Liang X, Hall JW, Yang J, Yan M, Doll K, et al (2011) Identification of single nucleotide polymorphisms associated with hyperproduction of alpha-toxin in *Staphylococcus aureus* . PLoS One 6: e18428. 21494631 10.1371/journal.pone.0018428 PMC3072997

[19] Bubeck Wardenburg J, Bae T, Otto M, Deleo FR, Schneewind O (2007) Poring over pores: alpha-hemolysin and Panton-Valentine leukocidin in *Staphylococcus aureus* pneumonia. Nat Med 13: 1405–1406. 18064027 10.1038/nm1207-1405

[20] Bubeck Wardenburg J, Patel RJ, Schneewind O (2007) Surface proteins and exotoxins are required for the pathogenesis of *Staphylococcus aureus* pneumonia. Infect Immun 75: 1040–1044. 17101657 10.1128/IAI.01313-06 PMC1828520

[21] Inoshima I, Inoshima N, Wilke GA, Powers ME, Frank KM, et al (2011) A *Staphylococcus aureus* pore-forming toxin subverts the activity of ADAM10 to cause lethal infection in mice. Nat Med 17: 1310–1314. 21926978 10.1038/nm.2451 PMC3192248

[22] Tamura K, Nei M, Kumar S (2004) Prospects for inferring very large phylogenies by using the neighbor-joining method. Proc Natl Acad Sci U S A 101: 11030–11035. 15258291 10.1073/pnas.0404206101 PMC491989

[23] Vandenesch F, Kornblum J, Novick RP (1991) A temporal signal, independent of *agr*, is required for hla but not *spa* transcription in *Staphylococcus aureus* . J Bacteriol 173: 6313–6320. 1717437 10.1128/jb.173.20.6313-6320.1991 PMC208961

[24] Zielinska AK, Beenken KE, Joo HS, Mrak LN, Griffin LM, et al (2011) Defining the strain-dependent impact of the Staphylococcal accessory regulator (*sarA*) on the alpha-toxin phenotype of *Staphylococcus aureus* . J Bacteriol 193: 2948–2958. 21478342 10.1128/JB.01517-10 PMC3133183

[25] De’ath G, Fabricius KE (2000) Classification and regression trees: a powerful yet simple thechnique for ecological data analysis. Ecology. Ecology 81: 3178–3192.

[26] Morfeldt E, Taylor D, von Gabain A, Arvidson S (1995) Activation of alpha-toxin translation in *Staphylococcus aureus* by the trans-encoded antisense RNA, RNAIII. EMBO J 14: 4569–4577. 7556100 10.1002/j.1460-2075.1995.tb00136.x PMC394549

[27] Wilson GJ, Seo KS, Cartwright RA, Connelley T, Chuang-Smith ON, et al (2011) A novel core genome-encoded superantigen contributes to lethality of community-associated MRSA necrotizing pneumonia. PLoS Pathog 7: e1002271. 22022262 10.1371/journal.ppat.1002271 PMC3192841

[28] Montoya M, Gouaux E (2003) Beta-barrel membrane protein folding and structure viewed through the lens of alpha-hemolysin. Biochim Biophys Acta 1609: 19–27. 12507754 10.1016/s0005-2736(02)00663-6

[29] Walker B, Bayley H (1995) Key residues for membrane binding, oligomerization, and pore forming activity of staphylococcal alpha-hemolysin identified by cysteine scanning mutagenesis and targeted chemical modification. J Biol Chem 270: 23065–23071. 7559447 10.1074/jbc.270.39.23065

[30] DeLeo FR, Kennedy AD, Chen L, Bubeck Wardenburg J, Kobayashi SD, et al (2011) Molecular differentiation of historic phage-type 80/81 and contemporary epidemic *Staphylococcus aureus* . Proc Natl Acad Sci U S A 108: 18091–18096. 22025717 10.1073/pnas.1111084108 PMC3207694

[31] Castillo-Ramirez S, Harris SR, Holden MT, He M, Parkhill J, et al (2011) The impact of recombination on dN/dS within recently emerged bacterial clones. PLoS Pathog 7: e1002129. 21779170 10.1371/journal.ppat.1002129 PMC3136474

[32] Kuhn G, Francioli P, Blanc DS (2006) Evidence for clonal evolution among highly polymorphic genes in methicillin-resistant *Staphylococcus aureus* . J Bacteriol 188: 169–178. 16352833 10.1128/JB.188.1.169-178.2006 PMC1317586

[33] Novick RP (2003) Autoinduction and signal transduction in the regulation of staphylococcal virulence. Mol Microbiol 48: 1429–1449. 12791129 10.1046/j.1365-2958.2003.03526.x

[34] Thoendel M, Kavanaugh JS, Flack CE, Horswill AR (2011) Peptide signaling in the staphylococci. Chem Rev 111: 117–151. 21174435 10.1021/cr100370n PMC3086461

[35] Tavares A, Miragaia M, Rolo J, Coelho C, de Lencastre H (2013) High prevalence of hospital-associated methicillin-resistant *Staphylococcus aureus* in the community in Portugal: evidence for the blurring of community-hospital boundaries. Eur J Clin Microbiol Infect Dis 32: 1269–1283. 23604782 10.1007/s10096-013-1872-2

[36] Oliveira DC, Tomasz A, de Lencastre H (2001) The evolution of pandemic clones of methicillin-resistant *Staphylococcus aureus*: identification of two ancestral genetic backgrounds and the associated *mec* elements. Microb Drug Resist 7: 349–361. 11822775 10.1089/10766290152773365

[37] Roberts RB, de Lencastre A, Eisner W, Severina EP, Shopsin B, et al (1998) Molecular epidemiology of methicillin-resistant *Staphylococcus aureus* in 12 New York hospitals. MRSA Collaborative Study Group. J Infect Dis 178: 164–171. 9652436 10.1086/515610

[38] Aires-de-Sousa M, Correia B, de Lencastre H (2008) Changing patterns in frequency of recovery of five methicillin-resistant *Staphylococcus aureus* clones in Portuguese hospitals: surveillance over a 16-year period. J Clin Microbiol 46: 2912–2917. 18614664 10.1128/JCM.00692-08 PMC2546730

[39] Rolo J, Miragaia M, Turlej-Rogacka A, Empel J, Bouchami O, et al (2012) High Genetic Diversity among Community-Associated *Staphylococcus aureus* in Europe: Results from a Multicenter Study. PLoS One 7: e34768. 22558099 10.1371/journal.pone.0034768 PMC3338755

[40] Conceicao T, Tavares A, Miragaia M, Hyde K, Aires-de-Sousa M, et al (2010) Prevalence and clonality of methicillin-resistant *Staphylococcus aureus* (MRSA) in the Atlantic Azores islands: predominance of SCC*mec* types IV, V and VI. Eur J Clin Microbiol Infect Dis 29: 543–550. 20229224 10.1007/s10096-010-0892-4 PMC2854357

[41] Sa-Leao R, Santos Sanches I, Dias D, Peres I, Barros RM, et al (1999) Detection of an archaic clone of *Staphylococcus aureus* with low-level resistance to methicillin in a pediatric hospital in Portugal and in international samples: relics of a formerly widely disseminated strain? J Clin Microbiol 37: 1913–1920. 10325346 10.1128/jcm.37.6.1913-1920.1999 PMC84983

[42] Amorim ML, Aires de Sousa M, Sanches IS, Sa-Leao R, Cabeda JM, et al (2002) Clonal and antibiotic resistance profiles of methicillin-resistant *Staphylococcus aureus* (MRSA) from a Portuguese hospital over time. Microb Drug Resist 8: 301–309. 12523627 10.1089/10766290260469561

[43] Milheirico C, Oliveira DC, de Lencastre H (2007) Multiplex PCR strategy for subtyping the staphylococcal cassette chromosome *mec* type IV in methicillin-resistant *Staphylococcus aureus*: ‘SCC*mec* IV multiplex’. J Antimicrob Chemother 60: 42–48. 17468509 10.1093/jac/dkm112

[44] Richardson JF, Reith S (1993) Characterization of a strain of methicillin-resistant *Staphylococcus aureus* (EMRSA-15) by conventional and molecular methods. J Hosp Infect 25: 45–52. 7901274 10.1016/0195-6701(93)90007-m

[45] Oliveira DC, Milheirico C, Vinga S, de Lencastre H (2006) Assessment of allelic variation in the *ccrAB* locus in methicillin-resistant *Staphylococcus aureus* clones. J Antimicrob Chemother 58: 23–30. 16735433 10.1093/jac/dkl208

[46] Faria NA, Oliveira DC, Westh H, Monnet DL, Larsen AR, et al (2005) Epidemiology of emerging methicillin-resistant *Staphylococcus aureus* (MRSA) in Denmark: a nationwide study in a country with low prevalence of MRSA infection. J Clin Microbiol 43: 1836–1842. 15815005 10.1128/JCM.43.4.1836-1842.2005 PMC1081382

[47] Sanches IS, Ramirez M, Troni H, Abecassis M, Padua M, et al (1995) Evidence for the geographic spread of a methicillin-resistant *Staphylococcus aureus* clone between Portugal and Spain. J Clin Microbiol 33: 1243–1246. 7615735 10.1128/jcm.33.5.1243-1246.1995 PMC228138

[48] de Lencastre H, Chung M, Westh H (2000) Archaic strains of methicillin-resistant *Staphylococcus aureus*: molecular and microbiological properties of isolates from the 1960s in Denmark. Microb Drug Resist 6: 1–10. 10868802 10.1089/mdr.2000.6.1

[49] Crisostomo MI, Westh H, Tomasz A, Chung M, Oliveira DC, et al (2001) The evolution of methicillin resistance in *Staphylococcus aureus*: similarity of genetic backgrounds in historically early methicillin-susceptible and -resistant isolates and contemporary epidemic clones. Proc Natl Acad Sci U S A 98: 9865–9870. 11481426 10.1073/pnas.161272898 PMC55544

[50] McDougal LK, Steward CD, Killgore GE, Chaitram JM, McAllister SK, et al (2003) Pulsed-field gel electrophoresis typing of oxacillin-resistant *Staphylococcus aureus* isolates from the United States: establishing a national database. J Clin Microbiol 41: 5113–5120. 14605147 10.1128/JCM.41.11.5113-5120.2003 PMC262524

