# Supplementary figures and images for: Insights into Alpha-Hemolysin (Hla) Evolution and Expression among Staphylococcus aureus Clones with Hospital and Community Origin
Source: PLoS One. 2014 Jul 17;9(7):e98634. doi: 10.1371/journal.pone.0098634 (PMC4102472; doi:10.1371/journal.pone.0098634)

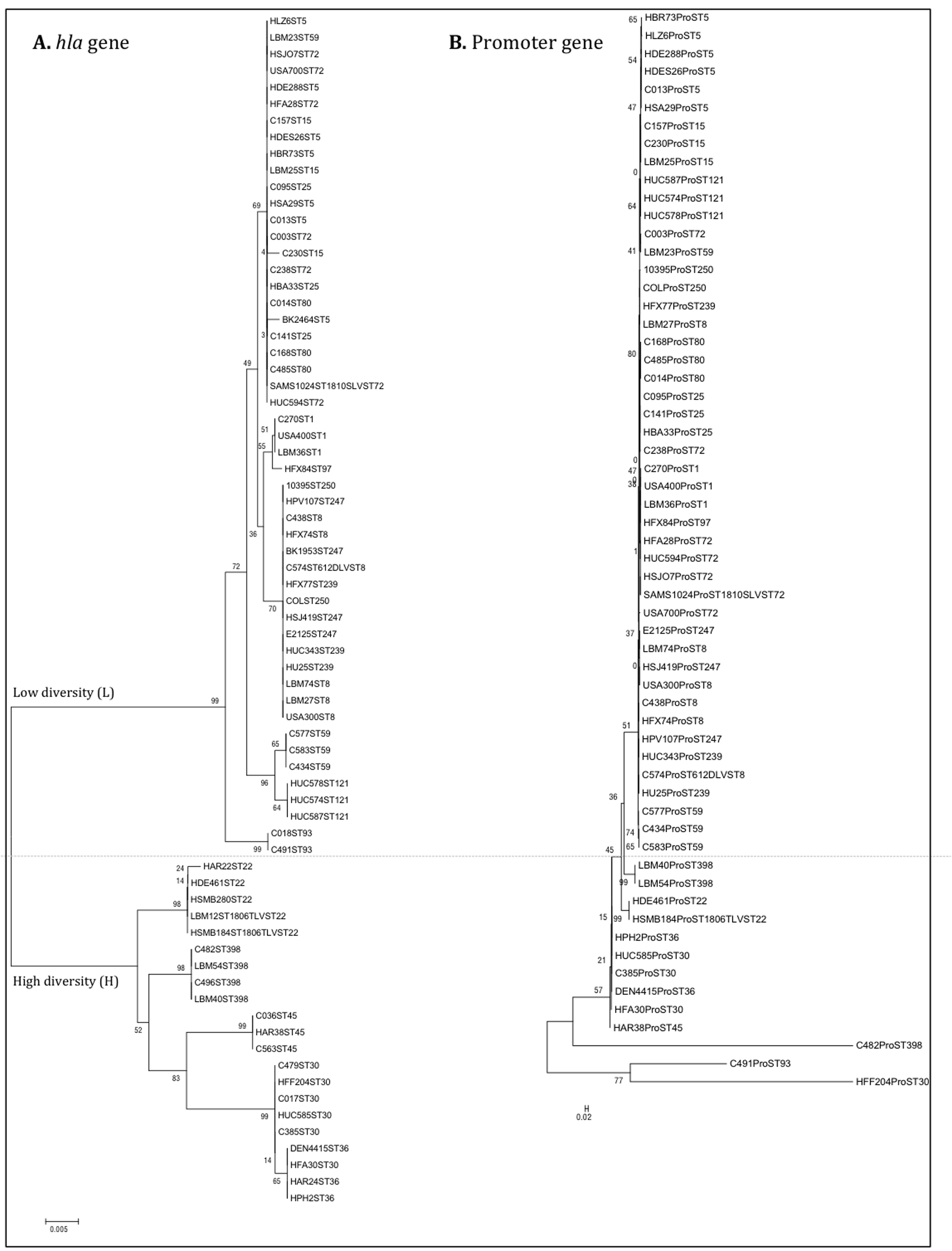

Supplement: Figure S1 — Phylogenetic trees of the hla gene, promoter gene and concatenated sequences of both. The tree was constructed using MEGA 5 with Neighbour-joining method and bootstrap values provided as percents over 1000 replications. Branch length values are indicated and the percentage of replicate trees (bootstrap test) are shown next to the branches. The dashed line indicates the separation of the two evolutionary branches (L and H). (TIF) [file pone.0098634.s001.tif]

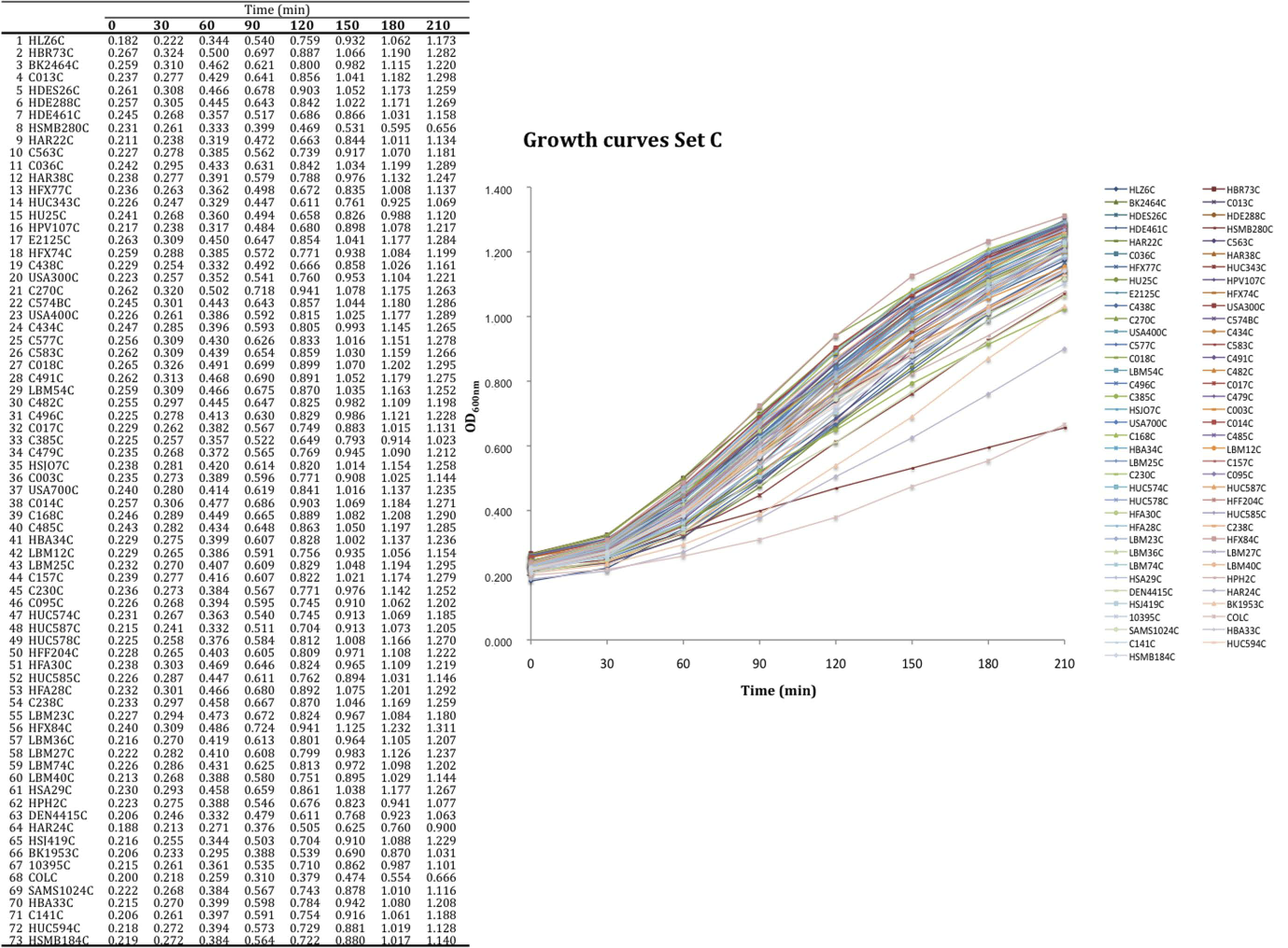

Supplement: Figure S2 — I. Growth curves for triplicates of each S. aureus strain – Set C. II. Growth curves for triplicates of each S. aureus strain – Set D. III. Growth curves for triplicates of each S. aureus strain – Set E. (TIFF) [file pone.0098634.s002.tif]

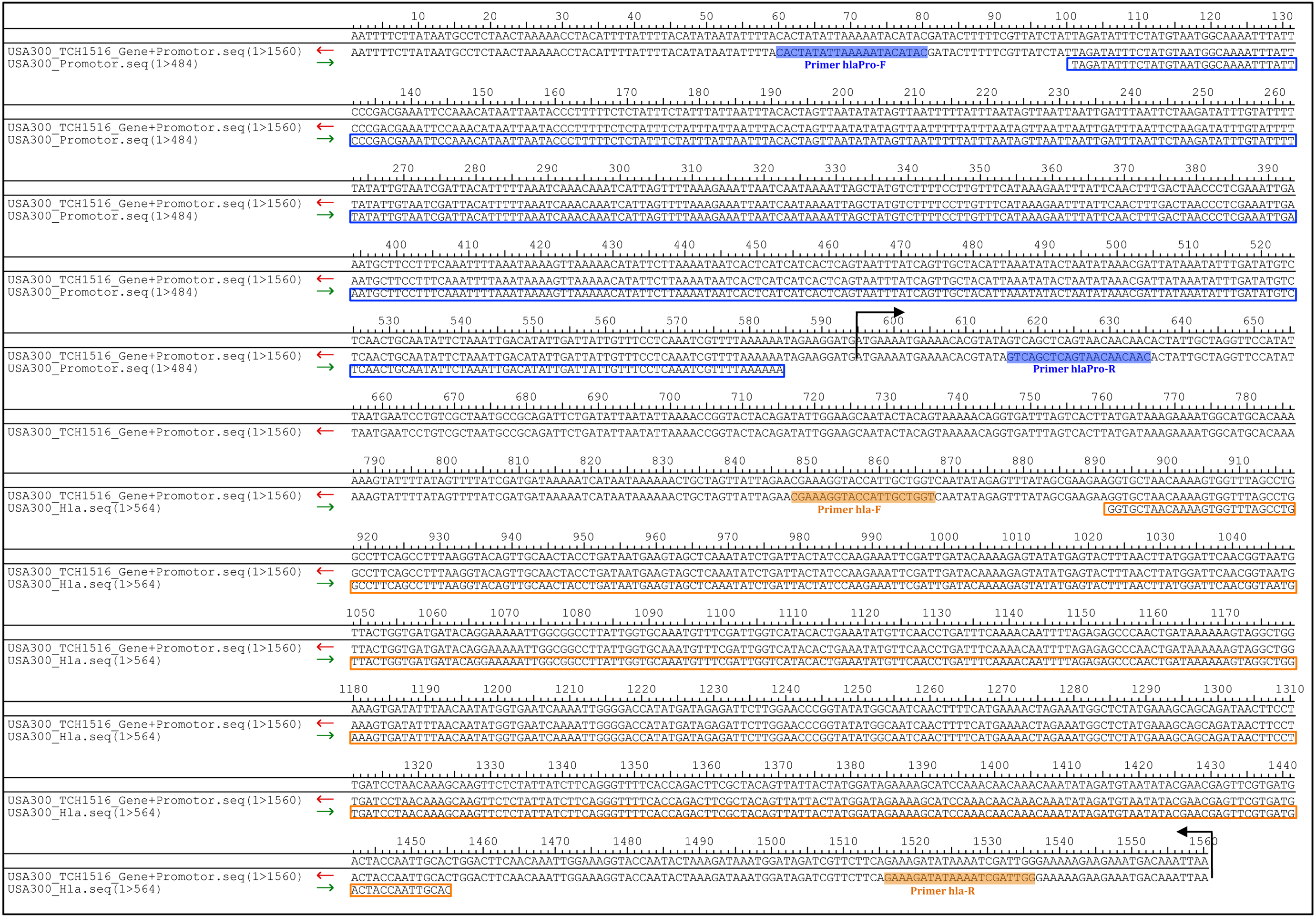

Supplement: Figure S3 — Internal sequences of hla promoter (highlighted blue) and hla gene (highlighted orange) used for analysis in this study. Primers used are highlighted. The sequence shown corresponds to the promoter and hla regions of USA300 strain from our collection blasted against USA300_TCH1516. (TIF) [file pone.0098634.s003.tif]
