# Supplementary material for: Insights into Alpha-Hemolysin (Hla) Evolution and Expression among Staphylococcus aureus Clones with Hospital and Community Origin
Source: PLoS One. 2014 Jul 17;9(7):e98634. doi: 10.1371/journal.pone.0098634 (PMC4102472; doi:10.1371/journal.pone.0098634)
Supplement: Table S1 — Molecular characterization of the 73 MRSA and MSSA strains included in this study [35]–[50]. (DOC) [file pone.0098634.s004.doc]

**Table S1.** Molecular characterization of the 73 MRSA and MSSA strains included in this study.

| **Nº** | **Isolate ID** | **Year** | **Country** (origin) | **MRSA/ MSSA** | **SCC*mec* type** | **SCCmec IV subtype** | **PVL** | **spa type** | **ST** | **Clone final classification** | **Reference** |
| --- | --- | --- | --- | --- | --- | --- | --- | --- | --- | --- | --- |
| 1 | HLZ6 | 2009 | Portugal | MRSA | II | n.a. | - | t002 | 5 | ST5 | 35 |
| 2 | BK2464 | 1990 | United Kindom | MRSA | II | n.a. | n.d. | t002 | 5 | NY/JP | 36, 37 |
| 3 | HBR73 | 2006 | Portugal | MRSA | II | n.a. | - | t067 | 5 | NY/JP | 38 |
| 4 | C013 | 2002 | Czech Republic | MRSA | VI | n.a. | + | t002 | 5 | Pediatric | 39 |
| 5 | HDES26 | 2007-2008 | Azores (PT) | MRSA | VI | n.a. | + | t062 | 5 | Pediatric | 40 |
| 6 | HDE288 | 1996 | Portugal | MRSA | VI | n.a. | - | t311 | 5 | Pediatric | 41 |
| 7 | HSA29 | 1992-1993 | Portugal | MSSA | n.a. | n.a. | n.d. | t002 | 5 | ST5 | 42 |
| 8 | HDE461 | 2006 | Portugal | MRSA | IV | n.d. | - | t022 | 22 | EMRSA15 | 38 |
| 9 | HAR22 | 1991 | United Kindom | MRSA | IV | IVh | - | t022 | 22 | EMRSA15 | 43, 44 |
| 10 | HSMB280 | 2009 | Portugal | MRSA | IV | IVh | - | t032 | 22 | EMRSA15 | 35 |
| 11 | LBM12 | 2009 | Portugal | MRSA | IV | IVh | - | t747 | 1806 | ST1806 (TLV ST22) | 35 |
| 12 | HSMB184 | 2009 | Portugal | MRSA | n.a. | n.a. | - | t5951 | 1806 | ST1806 (TLV ST22) | 35 |
| 13 | HPH2 | 2006 | Portugal | MRSA | II | n.a. | - | t018 | 36 | EMRSA16 | 38 |
| 14 | HAR24 | 1993 | United Kindom | MRSA | II | n.a. | n.d. | t018 | 36 | EMRSA16 | 45 |
| 15 | DEN4415 | 2001 | Denmark | MRSA | II | n.a. | n.d. | t021 | 36 | EMRSA16 | 46 |
| 16 | C563 | 2009 | Denmark | MRSA | IV | IV_NT_ | - | t015 | 45 | Berlin | 39 |
| 17 | C036 | 2004 | Czech Republic | MRSA | V | NA | - | t015 | 45 | Berlin | 39 |
| 18 | HAR38 | 1995 | Belgium | MRSA | IV | IVa | - | t004 | 45 | Berlin | 45, 36 |
| 19 | HFX77 | 2009 | Portugal | MRSA | III | n.a. | - | t037 | 239 | Brazilian | 35 |
| 20 | HUC343 | 2006 | Portugal | MRSA | IIIA | n.a. | - | t037 | 239 | Brazilian | 38 |
| 21 | HU25 | 1993 | Brazil | MRSA | IIIA | n.a. | - | t138 | 239 | Brazilian | 36 |
| 22 | BK1953 | 1995 | United Kindom | MRSA | IA | n.a. | n.d. | t051 | 247 | Iberian | 45,37 |
| 23 | HPV107 | 1992 | Portugal | MRSA | IA | n.a. | n.d. | t051 | 247 | Iberian | 47 |
| 24 | HSJ419 | 2006 | Portugal | MRSA | IA | n.a. | - | t725 | 247 | Iberian | 38 |
| 25 | E2125 | 1964 | Denmark | MRSA | I | n.a. | n.d. | t051 | 247 | Archaic | 48 |
| 26 | 10395 | 1961 | United Kindom | MRSA | I | n.a. | n.d. | t008 | 250 | Archaic | 49 |
| 27 | COL | 1965 | United Kindom | MRSA | I | n.a. | n.d. | t008 | 250 | Archaic | 36 |
| 28 | HFX74 | 2009 | Portugal | MRSA | IV | IVa | + | t008 | 8 | USA300 | 35 |
| 29 | USA300 | 1995-2003 | United States | MRSA | IV | IVa | + | t008 | 8 | USA300 | 50 |
| 30 | C438 | 2008 | Sweden | MRSA | IV | IVc | + | t024 | 8 | USA300 | 39 |
| 31 | C574B | 2009 | Denmark | MRSA | IV | IVd | - | t1257 | 612 | DLV ST8 | 39 |
| 32 | LBM27 | 2009 | Portugal | MSSA | n.a. | n.a. | - | t024 | 8 | USA300 like | 35 |
| 33 | LBM74 | 2009 | Portugal | MSSA | n.a. | n.a. | - | t008 | 8 | ST8 | 35 |
| 34 | C270 | 2009 | Romania | MRSA | IV | IVa | - | t1381 | 1 | USA400 like | 39 |
| 35 | USA400 | 1995-2003 | United States | MRSA | IV | IVa | + | t127 | 1 | USA400 | 50 |
| 36 | LBM36 | 2009 | Portugal | MSSA | n.a. | n.a. | - | t127 | 1 | USA400 like | 35 |
| 37 | C577 | 2009 | Denmark | MRSA | IV | IVa | + | t216 | 59 | Taiwan | 39 |
| 38 | C583 | 2009 | Denmark | MRSA | IV | IVa | - | t437 | 59 | Taiwan | 39 |
| 39 | C434 | 2009 | Sweden | MRSA | V | n.a. | + | t437 | 59 | Taiwan | 39 |
| 40 | C018 | 2002 | Czech Republic | MRSA | IV | IVa | + | t1819 | 93 | Queensland | 39 |
| 41 | C491 | no data | Netherlands | MRSA | IV | IVa | + | t202 | 93 | Queensland | 39 |
| 42 | LBM54 | 2009 | Portugal | MRSA | IV | n.d. | - | t011 | 398 | ST398 | 35 |
| 43 | C482 | no data | Netherlands | MRSA | IV | IVa | - | t011 | 398 | ST398 | 39 |
| 44 | C496 | no data | Netherlands | MRSA | VII | n.a. | - | t108 | 398 | ST398 | 39 |
| 45 | LBM40 | 2009 | Portugal | MSSA | n.a. | n.a. | - | t034 | 398 | ST398 | 35 |
| 46 | C017 | 2004 | Czech Republic | MRSA | IV | IVc | + | t019 | 30 | Southwesth Pacific | 39 |
| 47 | C385 | 2005 | Spain | MRSA | IV | IVc | + | t019 | 30 | Southwesth Pacific | 39 |
| 48 | C479 | 2005 | Netherlands | MRSA | IV | IVc | + | t019 | 30 | Southwesth Pacific | 39 |
| 49 | HSJO7 | 2009 | Portugal | MRSA | IV | n.d. | - | t148 | 72 | USA700 | 35 |
| 50 | USA700 | 1995-2003 | United States | MRSA | IV | IVa | + | t148 | 72 | USA700 | 50 |
| 51 | C003 | 2003 | Czech Republic | MRSA | IV | IVa | + | t791 | 72 | USA700 | 39 |
| 52 | SAMS1024 | 2009 | Portugal | MRSA | IV | IVa | - | t1346 | 1810 | ST1810 (SLV ST72) | 35 |
| 53 | HUC594 | 2009 | Portugal | MSSA | n.a. | n.a. | - | t148 | 72 | ST72 | 35 |
| 54 | HFA28 | 2009 | Portugal | MSSA | n.a. | n.a. | - | t126 | 72 | ST72 | 35 |
| 55 | C238 | 2008 | Czech Republic | MSSA | n.a. | n.a. | - | t3682 | 72 | ST72 | 39 |
| 56 | C168 | 2005 | Greece | MRSA | IV | IVc | + | t044 | 80 | European | 39 |
| 57 | C485 | no data | Netherlands | MRSA | IV | IVc | + | t044 | 80 | European | 39 |
| 58 | C014 | 2002 | Czech Republic | MRSA | IV | IVc | + | t131 | 80 | European | 39 |
| 59 | LBM25 | 2009 | Portugal | MSSA | n.a. | n.a. | - | t1509 | 15 | ST15 | 35 |
| 60 | C157 | 2009 | United Kindom | MSSA | n.a. | n.a. | - | t084 | 15 | ST15 | 39 |
| 61 | C230 | 2009 | Czech Republic | MSSA | n.a. | n.a. | - | t346 | 15 | ST15 | 39 |
| 62 | HBA33 | 2009 | Portugal | MSSA | n.a. | n.a. | - | t258 | 25 | ST25 | 35 |
| 63 | C095 | 2005 | Bulgaria | MSSA | n.a. | n.a. | + | t2909 | 25 | ST25 | 39 |
| 64 | C141 | 2009 | United Kindom | MSSA | n.a. | n.a. | - | t081 | 25 | ST25 | 39 |
| 65 | HBA34 | 2009 | Portugal | MRSA | IV | IV_NT_ | - | t308 | 121 | ST121 | 35 |
| 66 | HUC574 | 2009 | Portugal | MSSA | n.a. | n.a. | + | t435 | 121 | ST121 | 35 |
| 67 | HUC587 | 2009 | Portugal | MSSA | n.a. | n.a. | - | t159 | 121 | ST121 | 35 |
| 68 | HUC578 | 2009 | Portugal | MSSA | n.a. | n.a. | + | t284 | 121 | ST121 | 35 |
| 69 | HFF204 | 2005-2006 | Portugal | MSSA | n.a. | n.a. | + | t318 | 30 | ST30 | 38 |
| 70 | HFA30 | 2009 | Portugal | MSSA | n.a. | n.a. | - | t012 | 30 | ST30 | 35 |
| 71 | HUC585 | 2009 | Portugal | MSSA | n.a. | n.a. | - | t342 | 30 | ST30 | 35 |
| 72 | LBM23 | 2009 | Portugal | MSSA | n.a. | n.a. | - | t100 | 9 | ST9 | 35 |
| 73 | HFX84 | 2009 | Portugal | MSSA | n.a. | n.a. | - | t267 | 97 | ST97 | 35 |

n.a. not apply; n.d. not determined; Y yes; N no; NT non typable; MSSA methicillin-susceptible *S. aureus*; MRSA methicillin-resistant *S. aureus*; (-) negative; (+) positive; TLV triple locus variant.
